# Knockdown of TRPM7 prevents tumor growth, migration, and invasion through the Src, Akt, and JNK pathway in bladder cancer

**DOI:** 10.1186/s12894-020-00714-2

**Published:** 2020-09-09

**Authors:** Eun Hye Lee, So Young Chun, Bomi Kim, Bo Hyun Yoon, Jun Nyung Lee, Bum Soo Kim, Eun Sang Yoo, Sangkyu Lee, Phil Hyun Song, Tae Gyun Kwon, Yun-Sok Ha

**Affiliations:** 1 Joint Institute for Regenerative Medicine, Kyungpook National University Hospital, Daegu, Republic of Korea; 2 BioMedical Research Institute, Kyungpook National University Hospital, Daegu, Republic of Korea; 3 Department of Urology, School of Medicine, Kyungpook National University, Daegu, Republic of Korea; 4 Department of Urology, School of Medicine, Kyungpook National University, Kyungpook National University Chilgok Hospital, Daegu, Republic of Korea; 5 Department of Urology, School of Medicine, Kyungpook National University, Kyungpook National University Hospital, Daegu, Republic of Korea; 6 BK21 Plus KNU Multi-Omics Based Creative Drug Research Team, College of Pharmacy, Research Institute of Pharmaceutical Sciences, Kyungpook National University, Daegu, Republic of Korea; 7 Department of Urology, Yeungnam University College of Medicine, Daegu, Republic of Korea

**Keywords:** Bladder cancer, TRPM7, Src, Akt, JNK

## Abstract

**Background:**

Bladder cancer (BC) is one of the most common malignancies of the urinary tract. The role of transient receptor potential melastatin 7 (TRPM7) in BC remains unclear. The aim of this study was to investigate the function and signal transduction pathway of TRPM7 in BC.

**Methods:**

T24 and UMUC3 cells were used to evaluate the molecular mechanism of TRPM7 by immunoblot analysis. Small interfering RNA was used to knockdown TRPM7, and the effect of silencing TRPM7 was studied by wound healing, migration, and invasion assays in T24 and UMUC3 cells. Xenograft model study was obtained to analyze the effect of TRPM7 inhibition in vivo.

**Results:**

Silencing of TRPM7 decreased the migration and invasion ability of T24 and UMUC3 cells. The phosphorylation of Src, Akt, and JNK (c-Jun N-terminal kinase) was also suppressed by TRPM7 silencing. Src, Akt, and JNK inhibitors effectively inhibited the migration and invasion of T24 and UMUC3 cells. In addition, the TRPM7 inhibitor, carvacrol, limited the tumor size in a xenograft model.

**Conclusion:**

Our data reveal that TRPM7 regulates the migration and invasion of T24 and UMUC3 cells via the Src, Akt, and JNK signaling pathway. Therefore, TRPM7 suppression could be a potential treatment for BC patients.

## Background

Bladder cancer (BC) is a critical public health issue, and is known as the most common cancer of the urinary tract and the ninth most common cause of cancer related death worldwide [1]. There are approximately 400,000 cases of BC and 150,000 deaths per year [2]. The incidence of BC rises with age and the incidence is three times greater in in men than women. The most common symptom of BC is hematuria without pain [3], and BC is commonly diagnosed by cystoscopy and cytology [4].

The pathologic type of BC can be divided into two groups: non-muscle invasive BC (NMIBC) and muscle invasive BC (MIBC) [5]. NMIBC includes Ta and T1 stages, which are a low grade non-invasive papillary tumors that penetrate the basement membrane without invading the muscle layer of the bladder wall. MIBC includes T2–T4 stages which invade the muscle layer of bladder wall [6]. Around 70% of BC patients are diagnosed with NMIBC; however, these patients show a high reoccurrence rate with occasionally progressed stages with muscle layer invasion within 5 years [7]. The reason for recurrence with muscle invasion remains unclear [3]. Most deaths from BC result from metastasis to other organs due to chemotherapy resistance [8], and the majority of MIBC cases have metastasis, migration, and invasion [9, 10]. Indeed, approximately 25% of MIBC patients show metastasis, and patients with metastasis show poor prognosis [11]. Recently used pathological markers for prognosis require further study to clarify the most appropriate parameter [12]. Thus, there has been a sustained effort to understand the role of pathogical markers in predicting the therapeutic response and prognosis in BC patients [13]. In the clinic, immune check point inhibitors, such as Atezolizumab, have been approved and used in BC. However, the effectiveness is still under investigation, and has followed cisplatin-based chemotherapy for the past decade [14].

TRPM7 is endogenously expressed in several human organs [15] and presents in a tetrameric form [16]. TRPM7 is a member of the transient receptor potential (TRP) channel family which is a non-selective cation channel family [17, 18]. Specifically, TRPM7 is a bifunctional protein that is well known as an essential regulator of Ca^2+^ and Mg^2+^ homeostasis [17]. TRPM7 is a protein kinase that has crucial roles in regulating diverse cellular processes including cell proliferation, adhesion, migration, and survival by phosphorylating itself [19, 20]. As TRPM7 is highly involved in many types of cancers, research onTRPM7 related mechanisms is actively underway. TRPM7 is highly expressed in both glioblastoma [21] and pancreatic adenocarcinoma [22], and has been reported to be associated with tumor migration and invasion [20, 23, 24]. Indeed, somatic mutation of TRPM7 has been found in breast carcinoma [25], colon carcinoma [26], ovarian cancer, and gastric carcinoma [25]. In previous studies, TRPM7 has been reported to regulate cell migration and invasion through the MAPK pathway, as well as the PI3K/Akt, ERK, and JNK pathways in cancer [27–29].

However, the function and the mechanism of TRPM7 in BC is not clearly understood and further studies are required to fully elucidate its role. In the current study, by exploring the role of TRPM7 and its underlying mechanisms using BC cell lines, we suggest TRPM7 as a beneficial treatment for BC patients. In addition, we evaluated whether the TRPM7 inhibitor, carvacrol, inhibited tumor size in a xenograft model.

## Methods

### Reagents

Anti-phospho-Src, anti-Src, anti-phospho-Akt, anti-Akt, anti-phospho-Erk, anti-Erk, anti-phospho-JNK, anti-JNK, anti-phospho-p38, anti-p38, and anti-TRPM7 antibodies were obtained from Cell Signaling Technology (Beverly, MA, USA). Carvacrol, dimethyl sulfoxide (DMSO), crystal violet, cell migration kit, and cell invasion kit were purchased from Sigma-Aldrich (St. Louis, MO, USA).

### Bladder cancer cell lines

T24, J82, UMUC3, 5637, and HT1376 cell lines were purchased from American Type Culture Collection. Each cell line was cultured in RPMI 1640, DMEM high glucose, and MEM alpha media containing 10% FBS and 100 U/ml penicillin-streptomycin (Gibco, Waltham, MA, USA) and incubated at 37 °C in a humidified 5% CO_2_ atmosphere.

### siRNA treatment

Cells were plated at 2 × 10^5^ cells per well. AccuTarget Predesigned siRNAs specific for human TRPM7, and scramble siRNAs purchased from Bioneer (Daejeon, Korea) were used to knockdown TRPM7 expression. The sequences for human TRPM7 siRNA were as follows: Fwd 5′-GUC UUG CCA UGA AAU ACU CUU-3′ and Rev. 5′GAG UAU UUC AUG GCA AGA CUU-3′ (siRNA #1), and Fwd 5′-AGG AGA AGA UGC AAU UAA ATT-3′ and Rev. 5′-UUU AAU UGC AUC UUC UCC UAG-3′ (siRNA #2). The negative control (NC) group was treated with a non-targeted sequence, and the sequences for NC were as follows: Fwd 5′- UUC UCC GAA CGU GUC ACG UTT-3′ and Rev. 5′-ACG UGA CAC GUU CGG AGA ATT-3′. For transient transfection, Lipofectamine® RNAiMAX (13778–150; Invitrogen, Carlsbad, CA, USA) was used with siRNA (2–200 pmol/μL) to treat the cells for 6 h. Following treatment, the media was changed to a transfection reagent free media containing 10% FBS, and incubated for 48 h at 37 °C in a humidified 5% CO_2_ atmosphere.

### Immunoblot analysis

Treated cells were lysed using RIPA buffer as described previously [30]. Protein samples were loaded into SDS-PAGE gels and transferred to nitrocellulose membrane. The primary antibodies were applied overnight at 4 °C at 1:1000 dilutions after blocking with 5% skim milk solution. The secondary antibodies were applied at 1:5000 for 2 h at room temperature. The bands were developed using ECL reagent (Advensta, Menlo Park, CA, USA), images were captured with a chemi-doc image analyzer (iBright 1500, Thermo Fisher Scientific), and the values were quantified with Image J software (Wayne Rasband, retired from NIH).

### Wound healing assay

T24 and UMUC3 cells were plated in 6-well plates, and the cell layer was scratched with a pipette tip. Cells were washed with phosphate buffered saline to remove cell debris. The scratched area was monitored photographically and imaged at 0 and 24 h using an Olympus CKX41 inverted microscope coupled with a digital imaging system.

### Migration and invasion assay

T24 and UMUC3 cells in serum free media were seeded in 8-μm pore size cell culture inserts (#353097, BD Falcon, Franklin Lakes, NJ, USA) and in Matrigel coated inserts (#354480, Corning, NY, USA) for migration and invasion, respectively. Media containing 10% FBS was added to the lower chamber. The cells were removed from the insert after 24 h incubation, and cells on the chamber were stained with crystal violet solution. The stained cells were dissolved in DMSO, and measured with a microplate spectrophotometer (wavelength 590 nm, BioTek Instruments, Winooski, VT, USA).

### Cell viability assay (MTT assay)

T24 and UMUC3 cells were seeded in 96-well plates at a density of 1 × 10^4^ per well and treated with different doses of carvacrol or siRNA. Cells were stained with filtered 1 mg/mL MTT solution for 4 h at 37 °C in a humidified 5% CO_2_ atmosphere. Stained cells were dissolved in DMSO after removing media and MTT solution. Absorbance was measured with a microplate spectrophotometer at 570 nm wavelength.

### In vivo analysis

All animal study protocols were approved by the Institutional Animal Ethics Committee of Yeungnam University, College of Medicine (YUMC-2017-024). All animals were kept in a controlled specific pathogen free environment under a 12 h light/dark cycle at a temperature of 25.0 °C ± 0.2 °C and humidity of 45% ± 2%. All mice were freely provided with food and water. UMUC3 cells were injected into Balb/c nude mice (*n* = 6, 6-week-old, 18-20 g, female). According to 3R (reduction, replacement, refinement) of Institutional Animal Care and Use Committee (IACUC), we chose the minimal number of animals used. For animal welfare, in case of movement disorder, eating disorder and weight loss (20% of normal weight) we euthanized the animals in CO_2_ gas. UMUC3 cells (5 × 10^5^) were subcutaneously injected in 200 μL PBS-Matrigel (1:1, Matrigel: PBS). When the tumor reached 4–6 mm in diameter, carvacrol was intraperitoneally injected at a 50 mg/mL concentration once a week for 4 weeks. The tumor size was measured twice a week, for 4 weeks. After 4 weeks from starting day of size measurement, mice were anesthetized with Zoletil 0.006 cc/10 g (30 mg/kg) and Rompun 0.004 cc/10 g (10 mg/kg) and euthanized in CO_2_ gas.

### Source of animals

Balb/c nude mice were purchased from Orient (Seoul, Korea).

### Immunohistochemistry stain

The tumor tissues were fixed in 10% formalin solution to produce paraffin blocks. The samples were sectioned at a 4 μm thickness and deparaffinized. Slides were heated in citrate buffer and 0.2% triton x solution for 30 mins and 10 mins, respectively, and 5% BSA solution was used as a blocking reagent. The primary antibody was incubated overnight at 4 °C, and the secondary antibody (Alexa Fluor 594 Goat anti-rabbit IgG, 1:1000 dilution, Abcam, Cambridge, MA, USA) was incubated for 1 h at room temperature. The slides were mounted with DAPI containing mounting solution (Vectashield, Vector Laboratories, Burlingame, CA, USA).

### Statistical analysis

One-way analysis of variance was used to assess differences among the treatment groups. The criterion for statistical significance was set at *p* < 0.05 or *p* < 0.01.

## Result

### TRPM7 knockdown suppresses bladder cancer cell viability

**Fig. 1:**
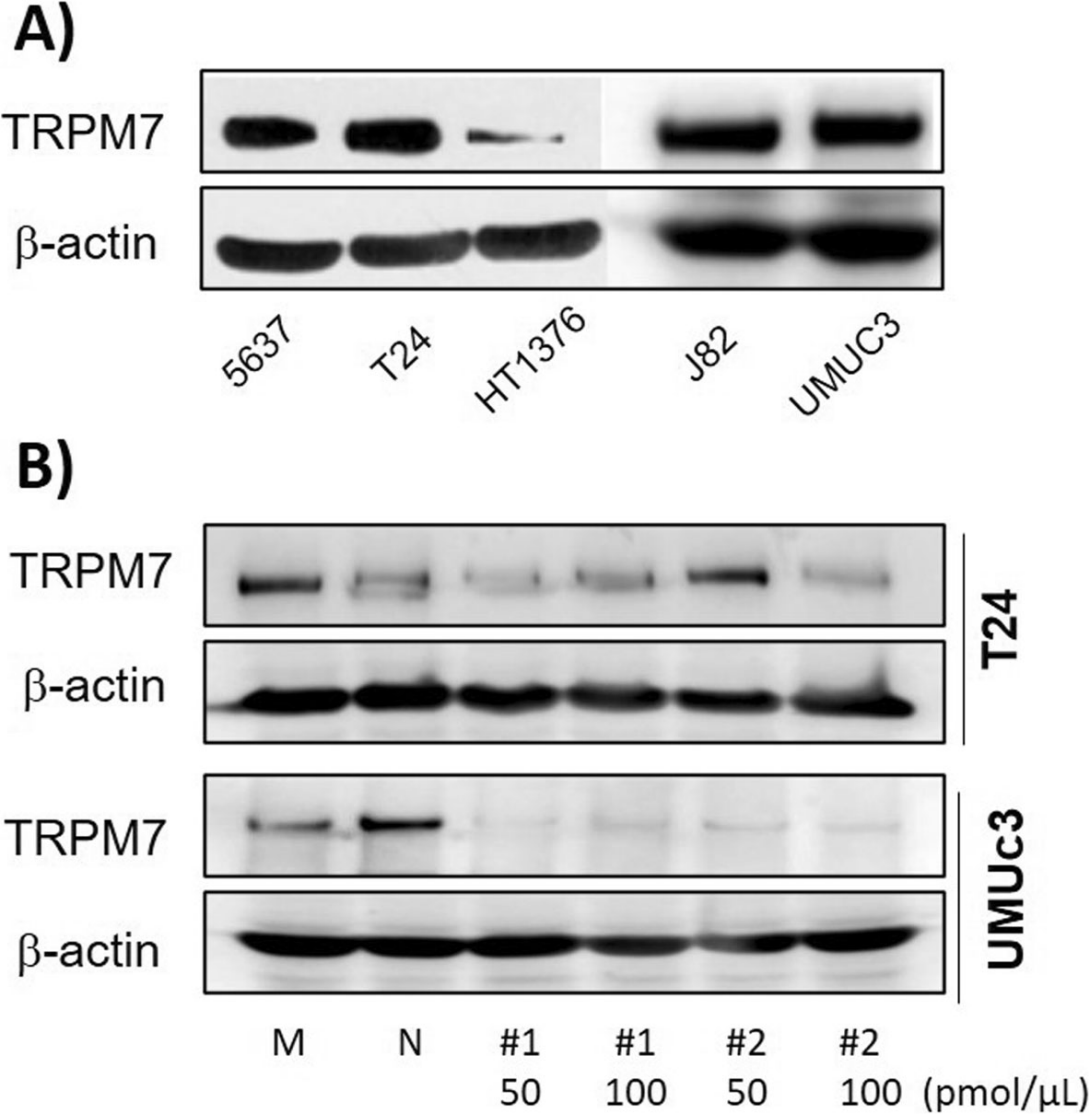
Protein expression of TRPM7 in bladder cancer cell lines by immunoblot analysis. **a** Protein expression of TRPM7 in 5637, T24, HT1376, J82, and UMUC3 cells. **b** Downregulation of TRPM7 in T24 and UMUC3 with different doses of siRNA. β-actin served as a loading control. M: Mock, N: Negative

**Fig. 2:**
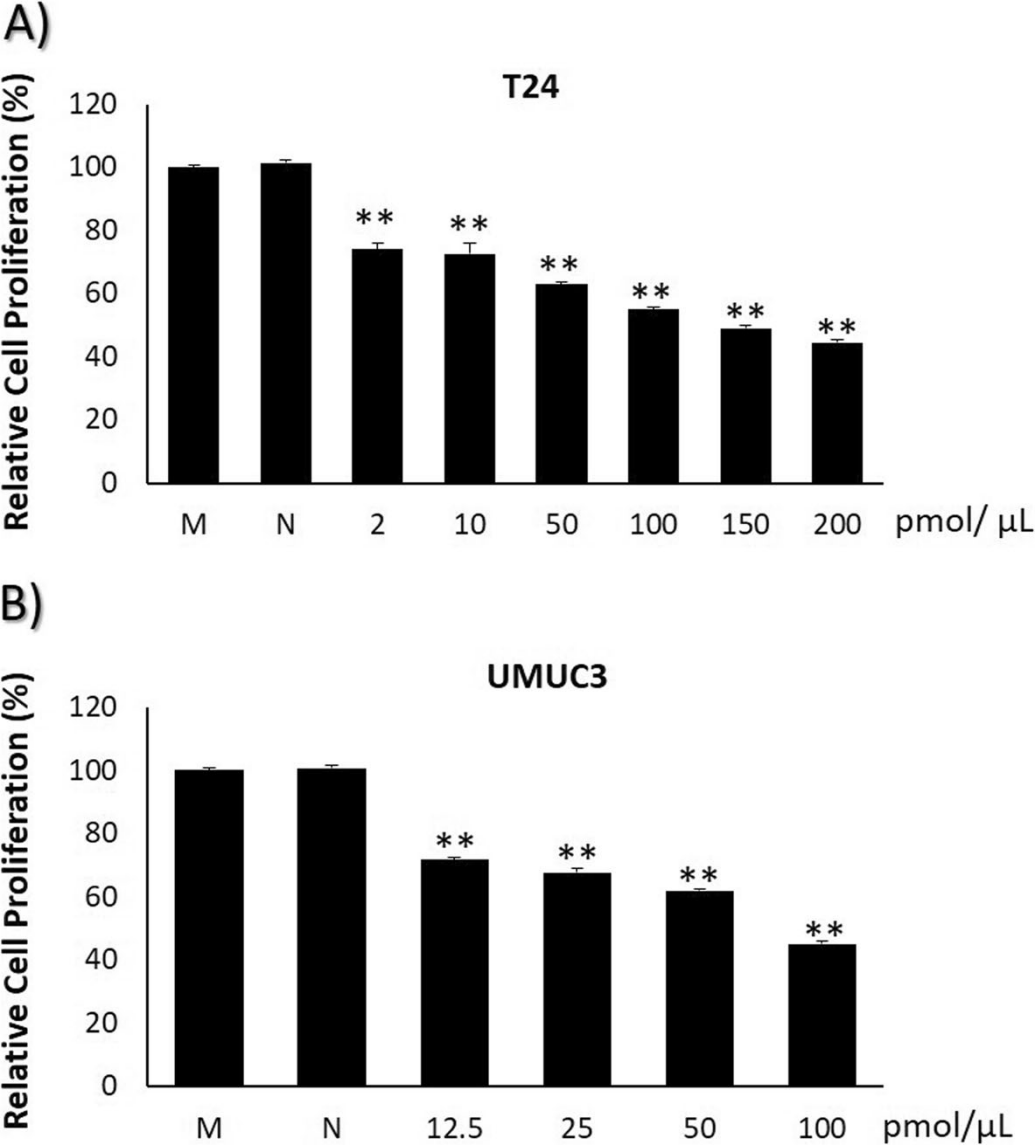
Effect of TRPM7 knockdown in bladder cancer cell proliferation. **a** T24 cells and (**b**) UMUC3 cells were treated with different doses of siRNA and incubated for 24 h. All data represent the means ± SD of three independent experiments (*p < 0.05 and **p < 0.01 between M and siRNA treated groups). M: Mock, N: Negative

In order to select the most suitable cell line, we checked the protein expression of TRPM7 in 5637, T24, HT1376, J82, and UMUC3 BC cell lines by immunoblot (Fig. 1a). Among these five cell lines, T24 and UMUC3 showed higher expression than HT1376. T24 and UMUC3 cell lines belong to transitional cell carcinoma, which is the most common type of bladder cancer, and T24 and UMUC3 showed high siRNA transfection efficacy. Next, we treated T24 and UMUC3 cells with two different sequences of siRNA in doses of 50 and 100 pmol/μL. The #2 sequence at dose of 100 pmol/μL siRNA significantly suppressed the expression of TRPM7 in T24 and UMUC3 cell lines, compared to mock and NC groups (Fig. 1b). Furthermore, the proliferation of T24 (Fig. 2a) and UMUC3 (Fig. 2b) cells was significantly inhibited by TRPM7 siRNA treatment, even at 2 pmol/μL, compared to the mock and NC groups. Full-length blots that presented in Fig. 1 are presented in Suppl. Fig. 4.

### TRPM7 knockdown reduces the motility of bladder cancer cells

**Fig. 3:**
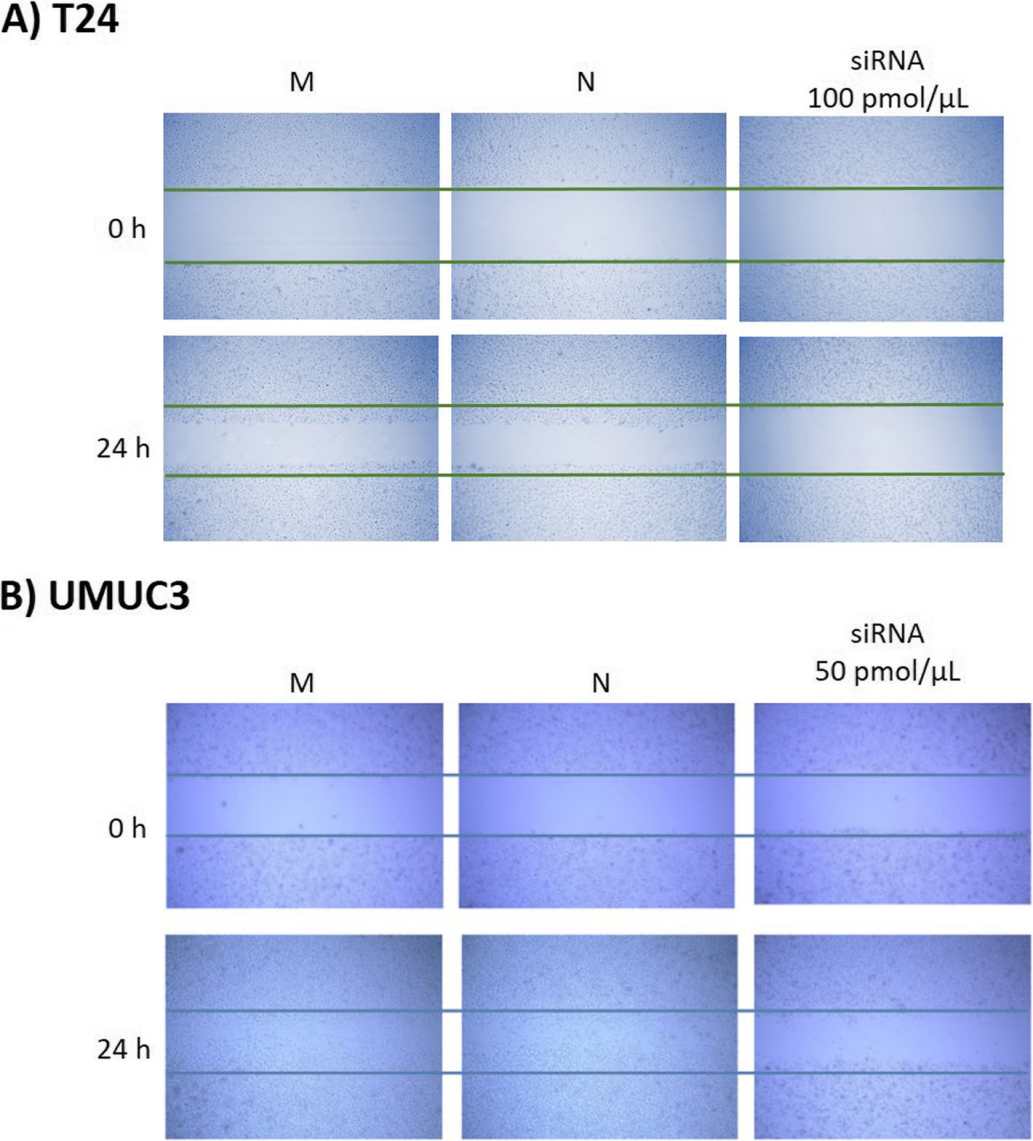
Effect of TRPM7 on cell migration in a wound healing assay. **a** T24 and (**b**) UMUC3 cells were treated with 100 pmol/μl and 50 pmol/μl and incubated for 24 h. M: Mock, N: Negative, h: Hour

**Fig. 4:**
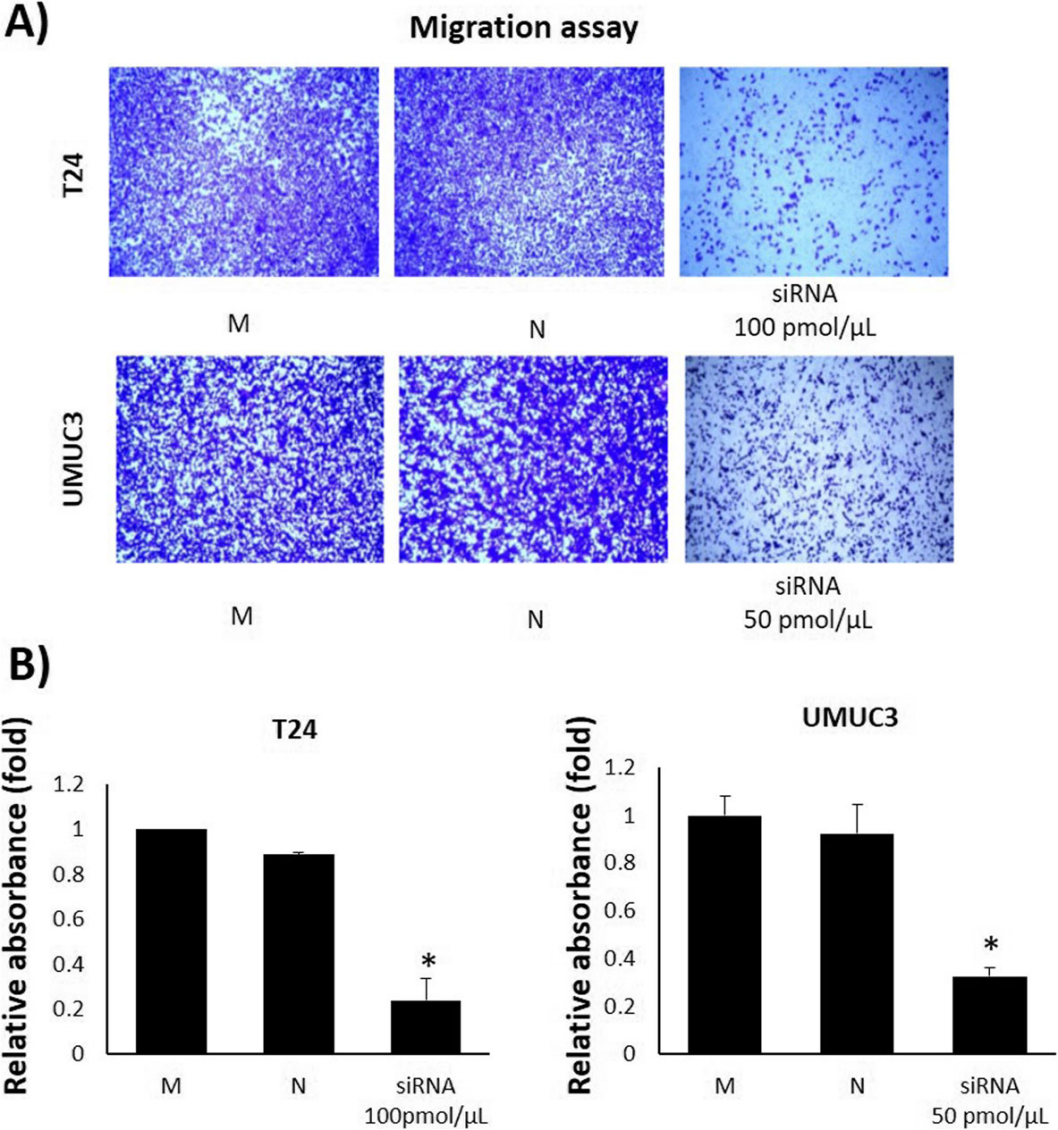
Effect of TRPM7 on cell migration in a cell permeable transwell. **a** Migration assay using a transwell with siRNA treatment. **b** Relative absorbance of migrated cells. The cells that passed through the 8 μm pore insert were stained with crystal violet solution. All data represent the means ± SD of three independent experiments (*p < 0.05 and **p < 0.01 between M and siRNA treated groups). M: Mock, N: Negative

**Fig. 5:**
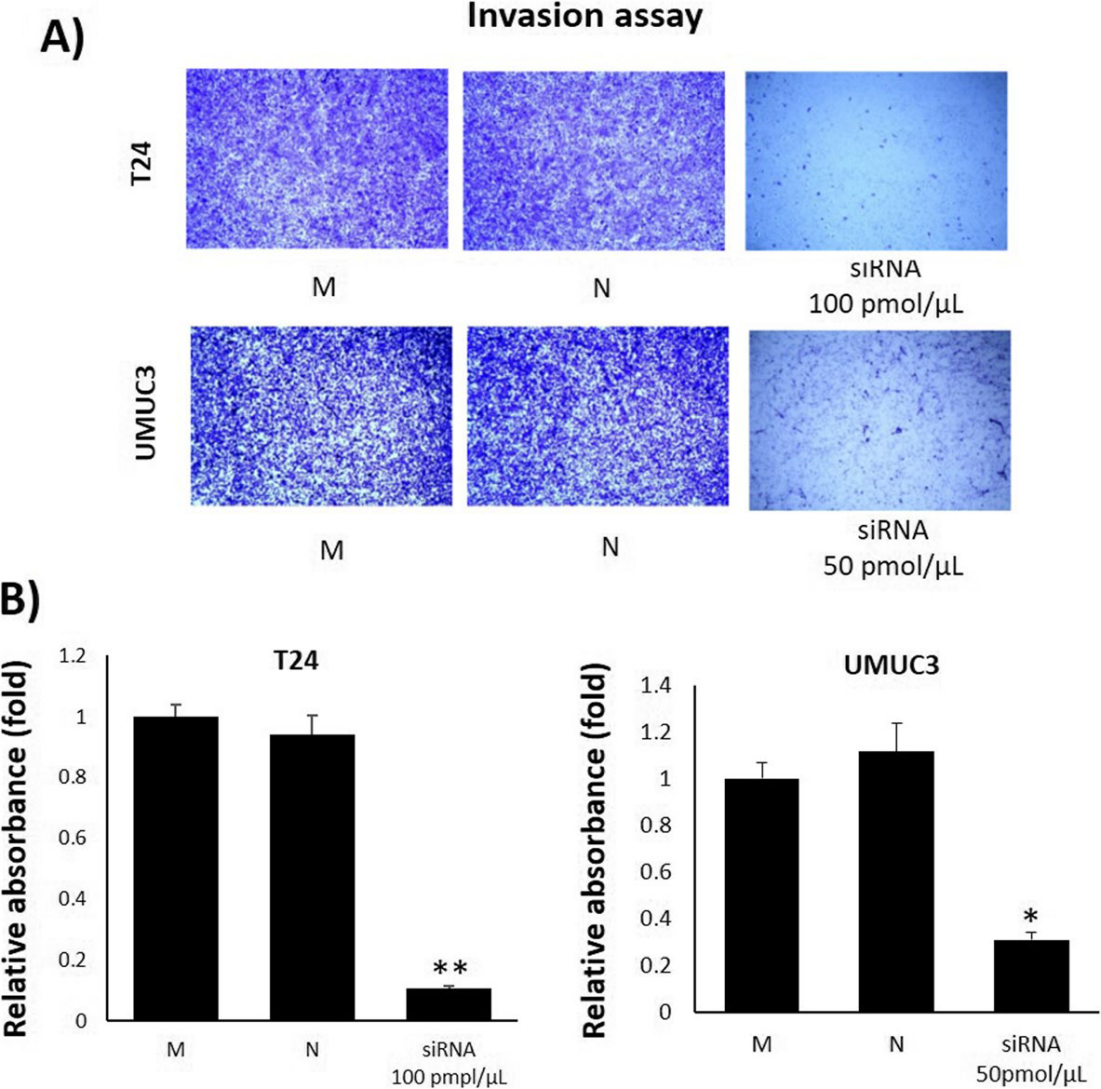
Effect of TRPM7 on Invasion. **a** Migration assay using a transwell with siRNA treatment. **b** Relative absorbance of migrated cells. The cells that passed through the 8-μm pore coated insert were stained with crystal violet solution. All data represent the means ± SD of three independent experiments (*p < 0.05 and **p < 0.01 between M and siRNA treated groups). M: Mock, N: Negative

A wound healing assay, migration assay, and invasion assay performed in order to analyze the effect of TRPM7 knockdown on cell motility. In the wound healing assay, siRNA treated (100 and 50 pmol/μL, non-lethal dose for each cell line) T24 and UMUC3 cells showed relatively wider wound gaps than non-treated groups following 24 h treatment (Fig. 3a and b). In the migration assay, siRNA treated T24 and UMUC3 cells showed significantly inhibited migration ability compared to non-treated groups (Fig. 4a and b). The invasion assay also showed significantly inhibited invasion in siRNA treated T24 and UMUC3 cells (Fig. 5a and b).

### TRPM7 regulates cell proliferation via Src, Akt, and JNK pathways

**Fig. 6:**
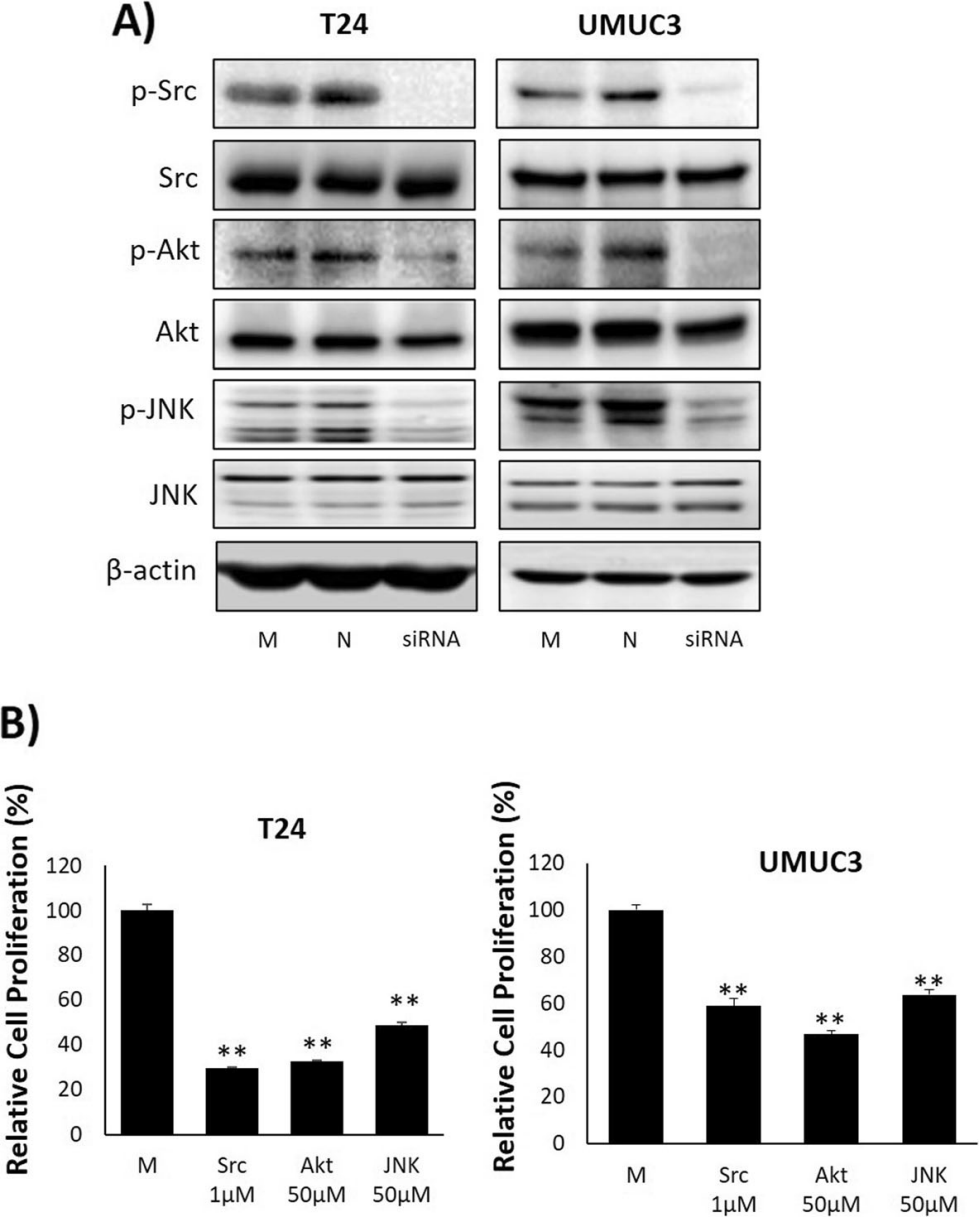
Effect of TRPM7 silencing in the Src, Akt, and JNK pathway. **a** Immunoblotting sowed that TRPM7 siRNA down-regulated protein expression of p-Src, p-Akt, and p-JNK. **b** Treatment of T24 and UMUC3 with Src, Akt, and JNK inhibitor induced significantly lowered cell proliferation compared to control. All data represent means ± SD of three independent experiments (*p < 0.05 and **p < 0.01 between M and inhibitor treated groups). M: Mock, N: Negative

The protein expression of Src, Akt and JNK (frequently related to cancer cell metastasis and invasion) was examined by immunoblot (Fig. 6). The results demonstrated that siRNA treated cells showed significantly reduced expression of p-Src, p-Akt, and p-JNK protein, while the total forms of Src, Akt, and JNK protein were not significantly changed by siRNA treatment (Fig. 6a). The association of these proteins with TRPM7 regulated cell proliferation was confirmed by treatment with the inhibitor of each protein (Fig. 6b). Both cell lines showed significantly decreased cell proliferation compared to the non-treated groups. Full-length blots that presented in Fig. 6 are presented in Suppl. Fig. 5.

### In vitro responses to TRPM7 inhibitor

To analysis cell responses to the TRPM7 inhibitor, the cell lines were treated with 100–500 μM of carvacrol. In cell proliferation analysis, the proliferation of T24 cells was significantly decreased in a dose dependent manner (from 300 μM) (Suppl. Fig. 1a) compared to UMUC3 (Suppl. Fig. 1b). In the cell migration (Suppl. Fig. 2) and invasion (Suppl. Fig. 3) assays, UMUC3 cells were significantly more sensitive to carvacrol (from 200 μM) compared to T24 cells.

### In vivo responses to TRPM7 inhibitor

**Fig. 7:**
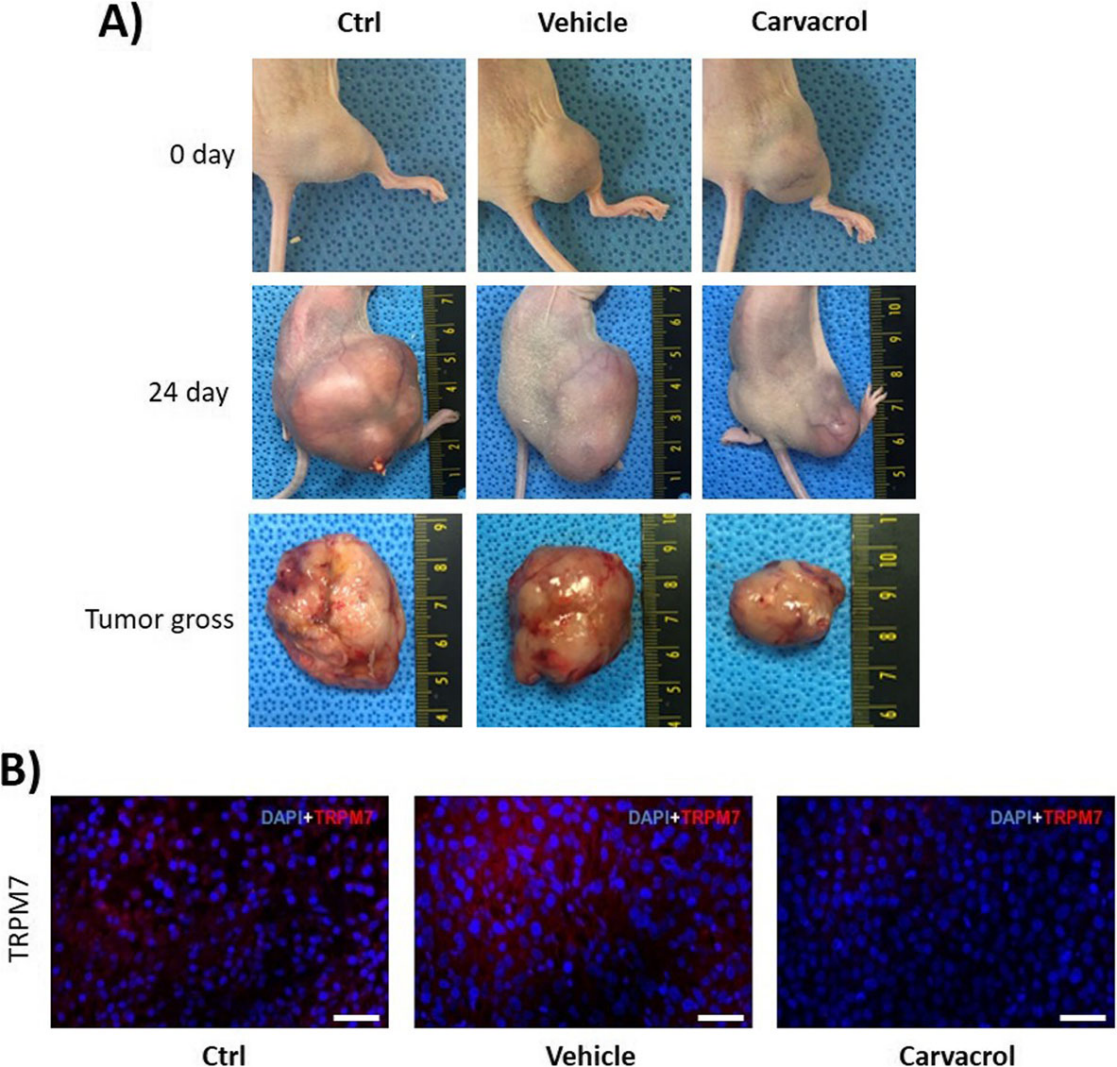
Effect of TRPM7 inhibitor on xenograft model. **a** UMUC3 cells were injected subcutaneously into Balb/c nude mice. Day 0 pictures were taken 2 weeks after UMUC3 cell injection, and Day 24 pictures were taken after the third injection of carvacrol. Tumor gross pictures were taken after the fourth injection of carvacrol. **b** Expression of TRPM7 in the control, vehicle, and carvacrol treated groups. Scale bar = 25 μm. Ctrl; no treat group, Vehicle; 1% DMSO injected group, Carvacrol; carvacrol injected group

Based on in vitro results, the in vivo condition was established using UMUC3 cells treated with 50 mg/mL carvacrol. At Day 24 after cell injection, the tumor volume was significantly reduced in the carvacrol treated group (468.45 ± 291.04 mm3) compared to the ctrl (4361.14 ± 2817.94 mm3) and vehicle (5176.45 ± 1603.25 mm3) groups (Fig. 7a). TRPM7 tumor expression was analyzed by IHC stain, wherein the carvacrol treated group showed significantly down-regulated expression of TRPM7 compared to the other groups (Fig. 7b).

## Discussion

Recently, TRPM7 has been reported to be implicated in carcinogenesis and has been considered as a potential target for diverse cancer treatment [27, 31]. TRPM7, one of the TRP member channels, is permeable to Ca^2+^ and Mg^2+^. Since Ca^2+^ is an important regulator of cell cycle and proliferation, TRPM7 regulation is considered to be critical to the biological function of cancer cells [31]. TRPM7 is reported to be overexpressed in human pulmonary adenocarcinoma, and pancreatic and prostate adenocarcinoma [18, 32]. Furthermore, downregulation of TRPM7 is known to inhibit the migration and invasion of breast cancer cells [27], while up-regulation has been shown to promote migration of lung cancer cells and vascular smooth muscle cells [23, 33].

Although numerous studies support that TRPM7 is involved in cancer cell migration and invasion [34–36], the precise understanding of the TRPM7 signaling pathway that modulates the molecular process of cancer cells remains unclear. According to previous studies, Src, Akt, and JNK are involved in cancer cell metastasis and invasion [37–40]. Indeed, Src was the first discovered oncogene, and it is able to activate caspase 8 by phosphorylation. Phosphorylated caspase-8 switches its function from pro-apoptotic to pro-migratory [41]. It has been reported that Src modulates the invasiveness of prostate cancer cells via regulating the E-cadherin/β-catenin complex [42]. Moreover, phosphorylated Akt serves as a cell proliferation boosting agent [43], while Akt has also been reported to promote invasion in fibrosarcoma and thyroid cancer cells [44, 45]. JNK is well known cancer regulating protein due to its action on oncogenes such as, Ras, c-fos, Met, and Bcr-Abl [46]. Furthermore, activated JNK induces the activation of apoptosis related Bcl-2 family proteins; thus, phosphorylated JNK acts as a pro-apoptotic signal in cancer cells [47]. In this study, we evaluated the TRPM7 mediated signaling pathway involved in BC cell lines, and demonstrated that silenced TRPM7 suppressed the protein expression of phosphorylation of Src, Akt, and JNK by immunoblot analysis. Based on this result, we can deduce TRPM7 regulates the Src, Akt, and JNK signaling pathway as a complex.

Our results also demonstrated that knockdown of TRPM7 by siRNA led to a decrease in proliferation of T24 and UMUC cells compared to the non-treated group, indicating that TRPM7 plays a key role in BC cell growth. In order to determine the effect of silenced TRPM7 on cell motility, we used T24 and UMUC3 cells in wound healing, migration, and invasion assays. Our results demonstrated that the TRPM7 silenced group showed a narrower area gap compared to the non-treated group in the wound healing assay, while the TRPM7 silenced group significantly suppressed the migration and invasion ability of BC cells. Thus, we were able to confirm that TRPM7 plays a crucial role in cell metastasis and invasion of BC cells.

To verify the effect of each protein (Src, Akt, and JNK) in cancer cell proliferation, we treated T24 and UMUC3 cells with inhibitors of Src, Akt, and JNK. As we expected, inhibition of Src, Akt, and JNK restricted cell proliferation. Based on the suppression of cell viability by Src, Akt, and JNK inhibition, we were able to derive that Src, Akt, and JNK play important roles in BC cell proliferation. In addition, we investigated the importance of TRPM7 in cancer growth by treatment with the TRPM7 inhibitor, carvacrol. In vitro, treatment of carvacrol showed decreased cell proliferation in a dose dependent manner compared to the non-treated group. In migration and invasion assays, as we expected, the migration and invasion capacity was lowest in carvacrol treated cells.

We evaluated the effect of TRPM7 inhibition in vivo by using carvacrol as a TRPM7 inhibitor in a xenograft model. Carvacrol injection reduced tumor size in nude mice, while the vehicle group showed similar tumor sizes to the control group. Furthermore, the carvacrol treated group had low TRPM7 protein expression by IHC. These in vivo results indicate that TRPM7 has a crucial role in tumor growth.

In previous studies regarding the function of TRPM7 in BC, the effect of TRPM7 downregulation induced BC cell apoptosis via the ERK1/2 pathway, while overexpressed TRPM7 promoted proliferation, migration, invasion, and tumor growth of BC [28]. Previous studies have demonstrated that TRPM7 downregulation increases reactive oxygen species, cell proliferation, migration, and invasion via elevated p-ERK1/2 and decreased PI3K/Akt protein expression [28]. According to other reports, overexpressed TRPM7 led to enhanced cell proliferation, migration, and invasion ability of BC cells [48]. In our study, we proved that TRPM7 mediates tumor growth, which is consistent with the findings of previously reports. As our limits, we proceeded cell cycle analysis; however, we excluded the results due to complicated understanding of the results. In addition, we were unable to investigate the expression of proteins related to proliferation, although we did examine the effect of TRPM7 knockdown in vivo, in order to extend the findings of our study.

## Conclusion

Taken together, this study provides the function and molecular mechanism of TRPM7 in BC. We demonstrated that knockdown of TRPM7 inhibits the migration and invasion of human BC cells via the Src, Akt, and JNK signaling pathway. Moreover, we demonstrated that suppression of TRPM7 inhibits tumor growth in a xenograft model. These results indicate that TRPM7 plays a crucial role in BC, and regulation of TRPM7 could be a potential treatment of BC.

## Supplementary information

**Additional file 1 Supplementary Fig. 1. Effect of carvacrol on T24 and UMUC3 cell viability.** (a) T24 and (b) UMUC3 cells were seeded in coated 96-well plates and grown until a confluency of 90%. Cells were treated with different doses (100, 200, 300, 400, 500 μM) of carvacrol and incubated for 24 h. All data represent the means ± SD of three independent experiments (*p < 0.05 and **p < 0.01 between control and carvacrol treated groups). Ctrl: Control.

**Additional file 2 Supplementary Fig. 2. Effect of carvacrol on bladder cancer cell migration.** (a) T24 and UMUC3 cells were seeded in cell transwells and treated with different doses (100, 200, 300, 400, 500 μM) of carvacrol before being incubated for 24 h. (b) The relative absorbance was measured in migrated cells stained with crystal violet at a wavelength of 590 nm. All data represent the means ± SD of three independent experiments (*p < 0.05 and **p < 0.01 between control and carvacrol treated groups). Ctrl: Control.

**Additional file 3 Supplementary Fig. 3. Effect of carvacrol on bladder cancer cell invasion.** (a) T24 and UMUC3 cells were seeded in the cell transwells and treated with different doses (100, 200, 300, 400, 500 μM) of carvacrol before being incubated for 24 h. (b) The relative absorbance was measured in invaded cells stained with crystal violet at a wavelength of 590 nm. All data represent the means ± SD of three independent experiments (*p < 0.05 and **p < 0.01 between control and carvacrol treated groups). Ctrl: Control.

**Additional file 4 Supplementary Fig. 4. Original western blot image of** Fig. 1a and b**.** (a) Beta-actin of J82 and UMUC3 in Fig. 1a. (b) TRPM7 of J82 and UMUC3 in Fig. 1a. (c) Beta-actin of siRNA treated T24 in Fig. 1b. (d) TRPM7 of siRNA treated T24 in Fig. 1b. (e) TRPM7 of siRNA treated UMUC3 in Fig. 1b. (f) Beta-actin of siRNA treated UMUC3 in Fig. 1b.

**Additional file 5 Supplementary Fig. 5. Original western blot image of** Fig. 6**a.** (a) Beta-actin of siRNA treated T24 in Fig. 6a. (b) p-Akt of siRNA treated T24 in Fig. 6a. (c) p-JNK of siRNA treated T24 in Fig. 6a. (d) p-Src of siRNA treated T24 in Fig. 6a. (e) t-Akt of siRNA treated T24 in Fig. 6a. (f) t-JNK of siRNA treated T24 in Fig. 6a. (g) t-Src of siRNA treated T24 in Fig. 6a. (h) Beta-actin of siRNA treated UMUC3 in Fig. 6a. (i) Beta-actin of siRNA treated UMUC3 in Fig. 6a. (j) p-Akt of siRNA treated UMUC3 in Fig. 6a. (k) p-Src of siRNA treated UMUC3 in Fig. 6a. (l) t-Akt of siRNA treated UMUC3 in Fig. 6a. (m) t-JNK of siRNA treated UMUC3 in Fig. 6a. (n) t-Src of siRNA treated UMUC3 in Fig. 6a. Full-length blots are presented in Suppl. Figs. 4 and 5.

**Additional file 6.** Fig. 1a beta-actin. Beta-actin of J82 and UMUC3 in Fig. 1a. Figure 1a TRPM7. TRPM7 of J82 and UMUC3 in Fig. 1a. Figure 1b beta-actin. Beta-actin of siRNA treated T24 in Fig. 1a. Fig. 1b, TRPM7_1. TRPM7 of siRNA treated T24 in Fig. 1b. Fig. 1b, TRPM7_2. TRPM7 of siRNA treated UMUC3 in Fig. 1b. Fig. 1b beta-actin. Beta-actin of siRNA treated UMUC3 in Fig. 1b. Figure 6a beta-actin-1. Beta-actin of siRNA treated T24 in Fig. 6a. Figure 6a p-Akt-1. p-Akt of siRNA treated T24 in Fig. 6a. Figure 6a p-JNK-1. p-JNK of siRNA treated T24 in Fig. 6a. Figure 6a p-Src-1. p-Src of siRNA treated T24 in Fig. 6a. Figure 6a t-Akt-1. t-Akt of siRNA treated T24 in Fig. 6a. Fig. 6a t-JNK-1. t-JNK of siRNA treated T24 in Fig. 6a. Fig. 6a t-Src-1. t-Src of siRNA treated T24 in Fig. 6a. Fig. 6a beta-actin-2. Beta-actin of siRNA treated UMUC3 in Fig. 6a. Fig. 6a p-Akt-2. p-Akt of siRNA treated UMUC3 in Fig. 6a. Fig. 6a p-JNK-2. p-JNK of siRNA treated UMUC3 in Fig. 6a. Fig. 6a p-Src. p-Src of siRNA treated UMUC3 in Fig. 6a. Fig. 6a t-Akt-2/ t-Akt of siRNA treated UMUC3 in Fig. 6a. Fig. 6a t-JNK-2/ t-JNK of siRNA treated UMUC3 in Fig. 6a. Fig. 6a t-Src. t-Src of siRNA treated UMUC3 in Fig. 6a.

## Data Availability

The datasets used and/or analyzed during the current study are available from the corresponding author on reasonable request.

## Abbreviations

BC: Bladder cancer
JNK: C-Jun N-terminal kinase
MIBC: Muscle invasive bladder cancer
NMIBC: Non-muscle invasive bladder cancer
TRP: Transient receptor potential
TRPM7: Transient receptor potential melastin 7
