# Supplementary figures and images for: Knockdown of TRPM7 prevents tumor growth, migration, and invasion through the Src, Akt, and JNK pathway in bladder cancer
Source: BMC Urol. 2020 Sep 9;20:145. doi: 10.1186/s12894-020-00714-2 (PMC7488071; doi:10.1186/s12894-020-00714-2)

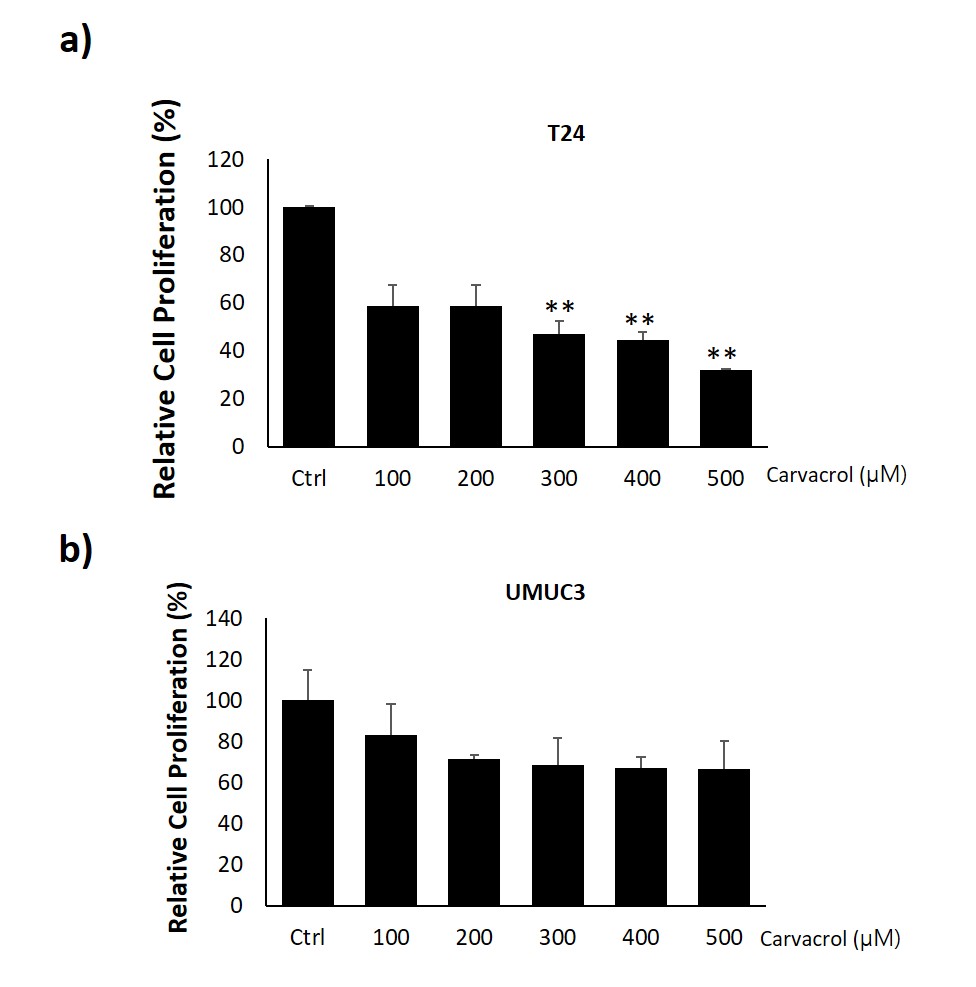

Supplement: Supplementary file 1 — Additional file 1 Supplementary Fig. 1. Effect of carvacrol on T24 and UMUC3 cell viability. (a) T24 and (b) UMUC3 cells were seeded in coated 96-well plates and grown until a confluency of 90%. Cells were treated with different doses (100, 200, 300, 400, 500 μM) of carvacrol and incubated for 24 h. All data represent the means ± SD of three independent experiments (*p < 0.05 and **p < 0.01 between control and carvacrol treated groups). Ctrl: Control. [file 12894_2020_714_MOESM1_ESM.jpg]

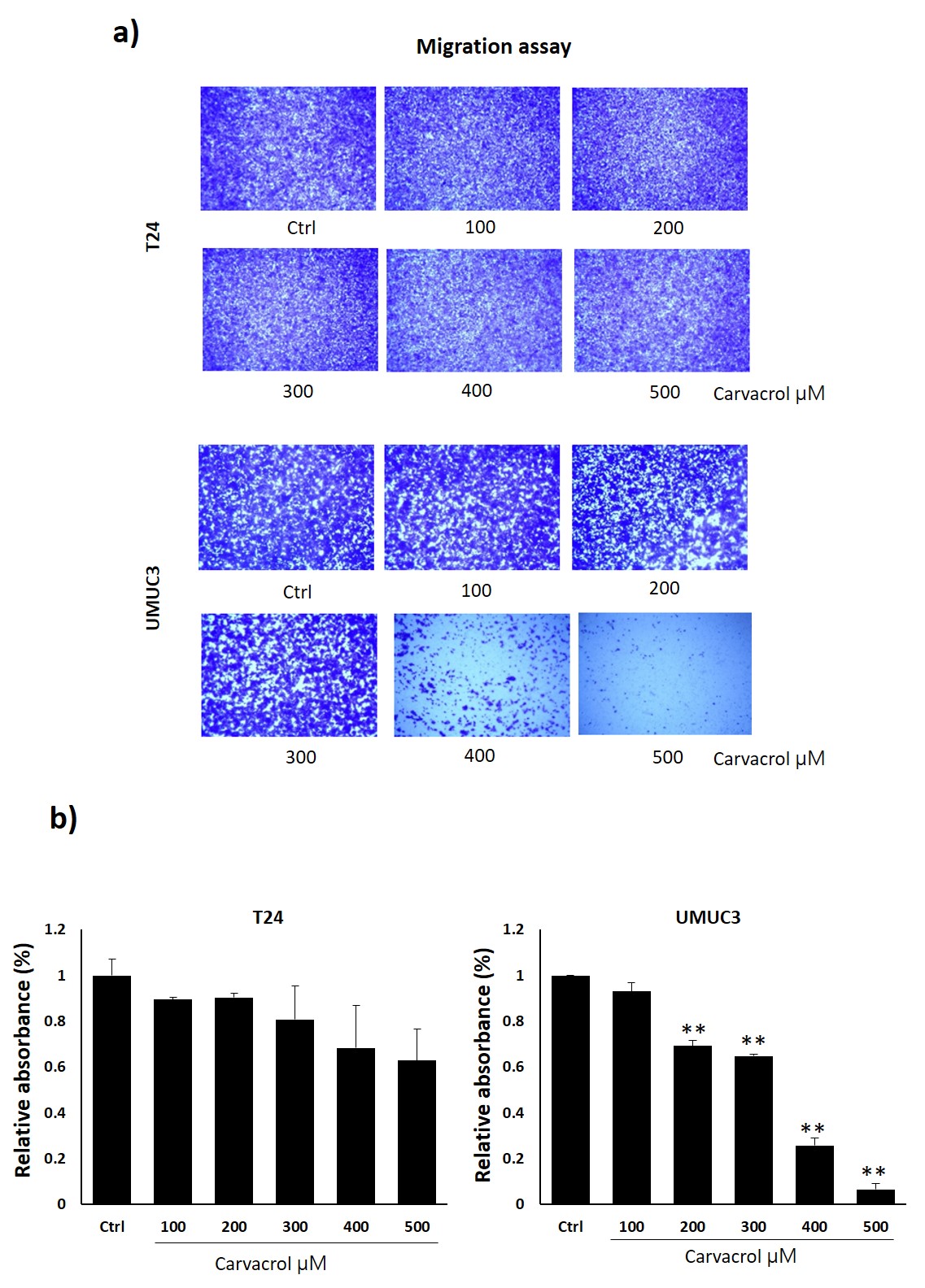

Supplement: Supplementary file 2 — Additional file 2 Supplementary Fig. 2. Effect of carvacrol on bladder cancer cell migration. (a) T24 and UMUC3 cells were seeded in cell transwells and treated with different doses (100, 200, 300, 400, 500 μM) of carvacrol before being incubated for 24 h. (b) The relative absorbance was measured in migrated cells stained with crystal violet at a wavelength of 590 nm. All data represent the means ± SD of three independent experiments (*p < 0.05 and **p < 0.01 between control and carvacrol treated groups). Ctrl: Control. [file 12894_2020_714_MOESM2_ESM.jpg]

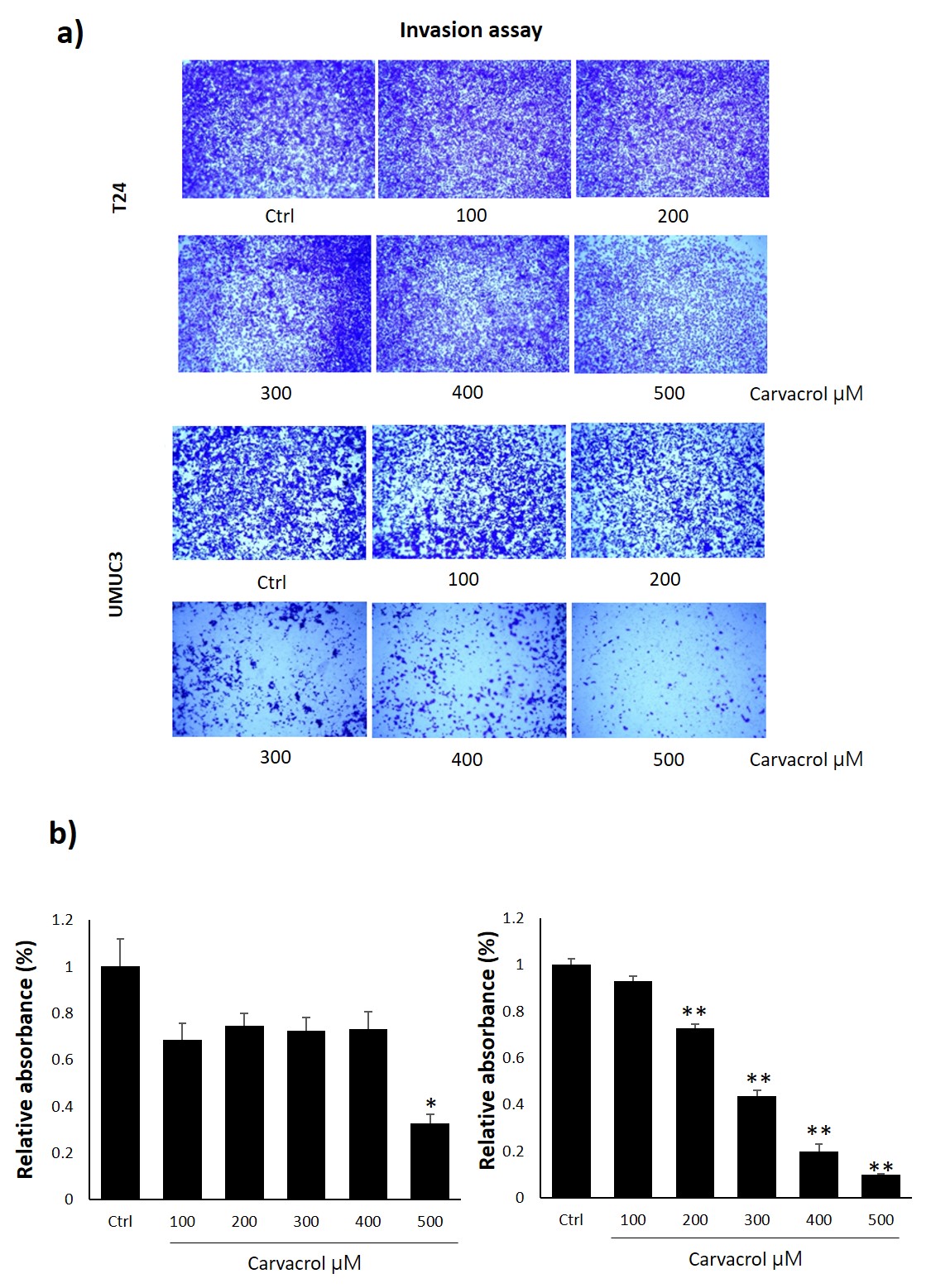

Supplement: Supplementary file 3 — Additional file 3 Supplementary Fig. 3. Effect of carvacrol on bladder cancer cell invasion. (a) T24 and UMUC3 cells were seeded in the cell transwells and treated with different doses (100, 200, 300, 400, 500 μM) of carvacrol before being incubated for 24 h. (b) The relative absorbance was measured in invaded cells stained with crystal violet at a wavelength of 590 nm. All data represent the means ± SD of three independent experiments (*p < 0.05 and **p < 0.01 between control and carvacrol treated groups). Ctrl: Control. [file 12894_2020_714_MOESM3_ESM.jpg]

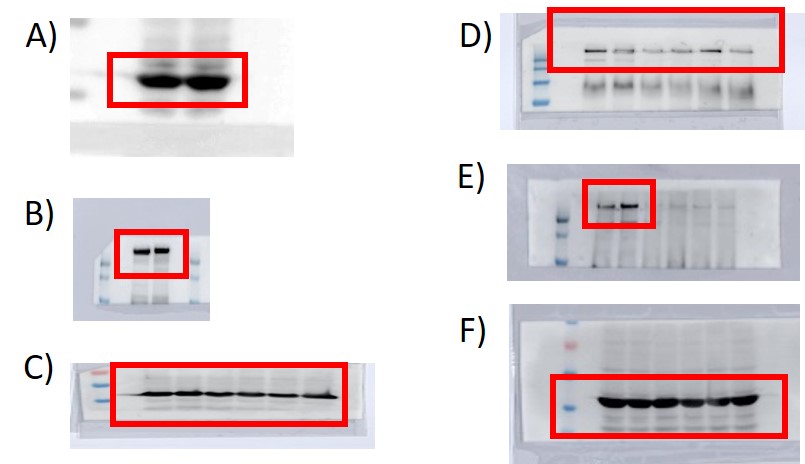

Supplement: Supplementary file 4 — Additional file 4 Supplementary Fig. 4. Original western blot image of Fig. 1a and b. (a) Beta-actin of J82 and UMUC3 in Fig. 1a. (b) TRPM7 of J82 and UMUC3 in Fig. 1a. (c) Beta-actin of siRNA treated T24 in Fig. 1b. (d) TRPM7 of siRNA treated T24 in Fig. 1b. (e) TRPM7 of siRNA treated UMUC3 in Fig. 1b. (f) Beta-actin of siRNA treated UMUC3 in Fig. 1b. [file 12894_2020_714_MOESM4_ESM.jpg]

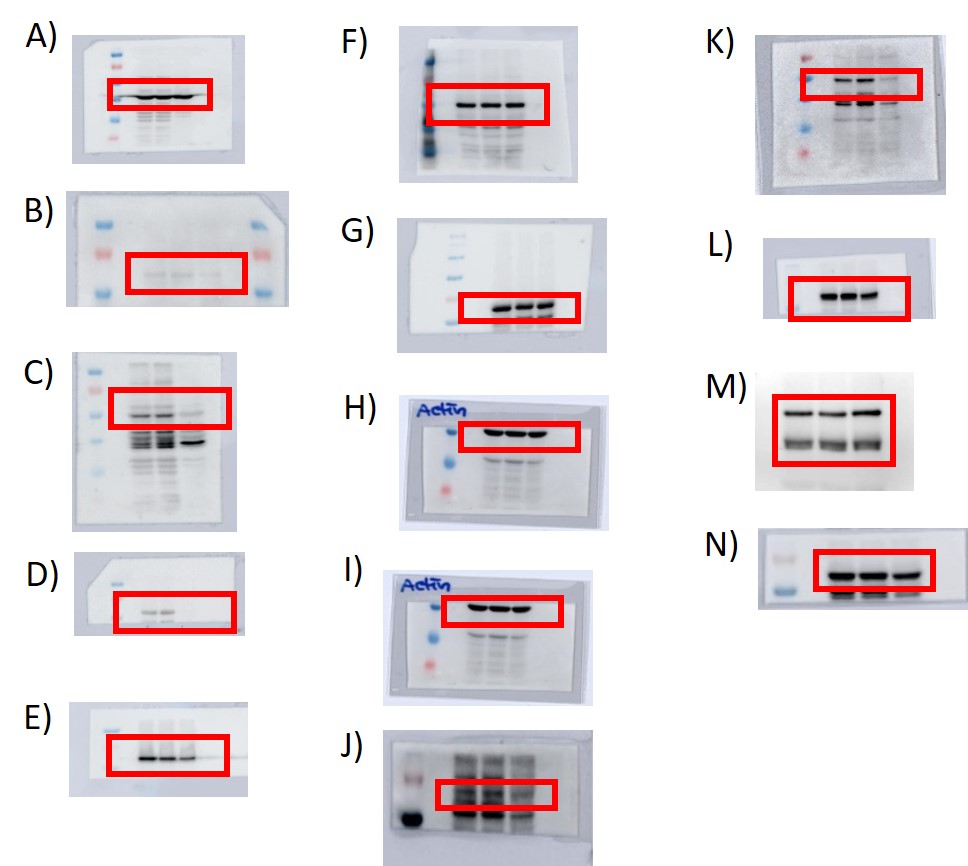

Supplement: Supplementary file 5 — Additional file 5 Supplementary Fig. 5. Original western blot image of Fig. 6a. (a) Beta-actin of siRNA treated T24 in Fig. 6a. (b) p-Akt of siRNA treated T24 in Fig. 6a. (c) p-JNK of siRNA treated T24 in Fig. 6a. (d) p-Src of siRNA treated T24 in Fig. 6a. (e) t-Akt of siRNA treated T24 in Fig. 6a. (f) t-JNK of siRNA treated T24 in Fig. 6a. (g) t-Src of siRNA treated T24 in Fig. 6a. (h) Beta-actin of siRNA treated UMUC3 in Fig. 6a. (i) Beta-actin of siRNA treated UMUC3 in Fig. 6a. (j) p-Akt of siRNA treated UMUC3 in Fig. 6a. (k) p-Src of siRNA treated UMUC3 in Fig. 6a. (l) t-Akt of siRNA treated UMUC3 in Fig. 6a. (m) t-JNK of siRNA treated UMUC3 in Fig. 6a. (n) t-Src of siRNA treated UMUC3 in Fig. 6a. Full-length blots are presented in Suppl. Figs. 4 and 5. [file 12894_2020_714_MOESM5_ESM.jpg]
