# Supplementary material for: Knockdown of TRPM7 prevents tumor growth, migration, and invasion through the Src, Akt, and JNK pathway in bladder cancer
Source: BMC Urol. 2020 Sep 9;20:145. doi: 10.1186/s12894-020-00714-2 (PMC7488071; doi:10.1186/s12894-020-00714-2)
Supplement: Supplementary file 6 — Additional file 6. Fig. 1a beta-actin. Beta-actin of J82 and UMUC3 in Fig. 1a. Figure 1a TRPM7. TRPM7 of J82 and UMUC3 in Fig. 1a. Figure 1b beta-actin. Beta-actin of siRNA treated T24 in Fig. 1a. Fig. 1b, TRPM7_1. TRPM7 of siRNA treated T24 in Fig. 1b. Fig. 1b, TRPM7_2. TRPM7 of siRNA treated UMUC3 in Fig. 1b. Fig. 1b beta-actin. Beta-actin of siRNA treated UMUC3 in Fig. 1b. Figure 6a beta-actin-1. Beta-actin of siRNA treated T24 in Fig. 6a. Figure 6a p-Akt-1. p-Akt of siRNA treated T24 in Fig. 6a. Figure 6a p-JNK-1. p-JNK of siRNA treated T24 in Fig. 6a. Figure 6a p-Src-1. p-Src of siRNA treated T24 in Fig. 6a. Figure 6a t-Akt-1. t-Akt of siRNA treated T24 in Fig. 6a. Fig. 6a t-JNK-1. t-JNK of siRNA treated T24 in Fig. 6a. Fig. 6a t-Src-1. t-Src of siRNA treated T24 in Fig. 6a. Fig. 6a beta-actin-2. Beta-actin of siRNA treated UMUC3 in Fig. 6a. Fig. 6a p-Akt-2. p-Akt of siRNA treated UMUC3 in Fig. 6a. Fig. 6a p-JNK-2. p-JNK of siRNA treated UMUC3 in Fig. 6a. Fig. 6a p-Src. p-Src of siRNA treated UMUC3 in Fig. 6a. Fig. 6a t-Akt-2/ t-Akt of siRNA treated UMUC3 in Fig. 6a. Fig. 6a t-JNK-2/ t-JNK of siRNA treated UMUC3 in Fig. 6a. Fig. 6a t-Src. t-Src of siRNA treated UMUC3 in Fig. 6a. [file 12894_2020_714_MOESM6_ESM.docx]

**Additional files**


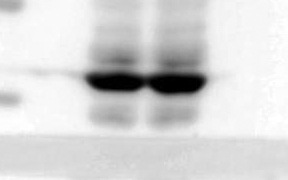


Title of data: Fig. 1A beta-actin

Description of data: Beta-actin of J82 and UMUC3 in Fig. 1A.


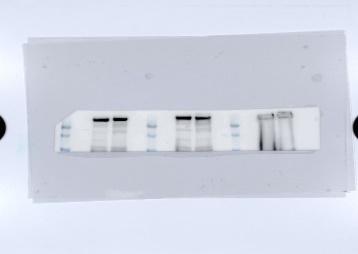


Title of data: Fig. 1A TRPM7

Description of data: TRPM7 of J82 and UMUC3 in Fig. 1A.


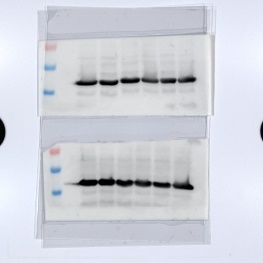


Title of data: Fig. 1B beta-actin

Description of data: Beta-actin of siRNA treated T24 in Fig. 1B.


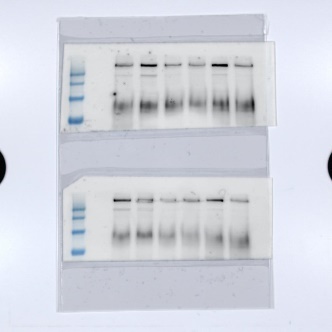


Title of data: Fig. 1B, TRPM7_1

Description of data: TRPM7 of siRNA treated T24 in Fig. 1B.


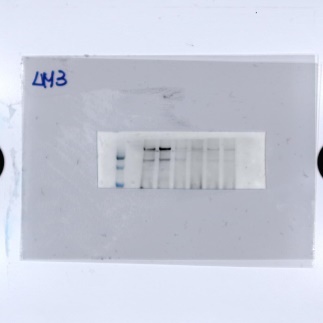


Title of data: Fig. 1B, TRPM7_2

Description of data: TRPM7 of siRNA treated UMUC3 in Fig. 1B.


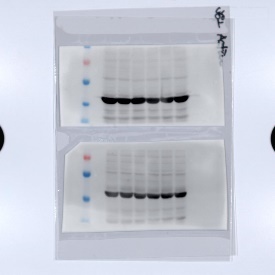


Title of data: Fig. 1B beta-actin

Description of data: Beta-actin of siRNA treated UMUC3 in Fig. 1B.


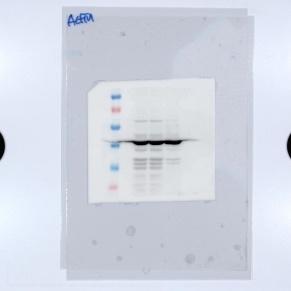


Title of data: Fig. 6A beta-actin-1

Description of data: Beta-actin of siRNA treated T24 in Fig. 6A.


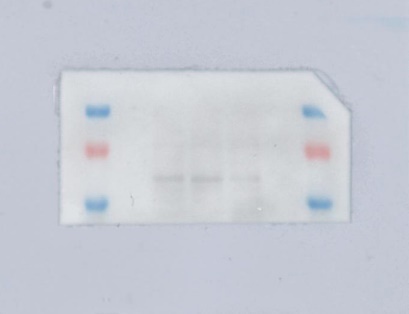


Title of data: Fig. 6A p-Akt-1

Description of data: p-Akt of siRNA treated T24 in Fig. 6A.


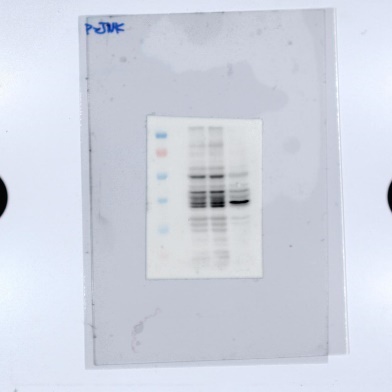


Title of data: Fig. 6A p-JNK-1

Description of data: p-JNK of siRNA treated T24 in Fig. 6A.


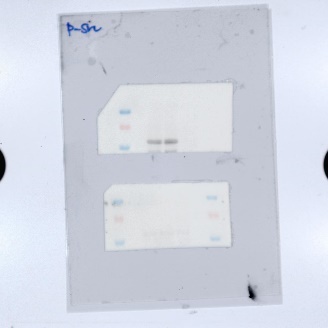


Title of data: Fig. 6A p-Src-1

Description of data: p-Src of siRNA treated T24 in Fig. 6A.


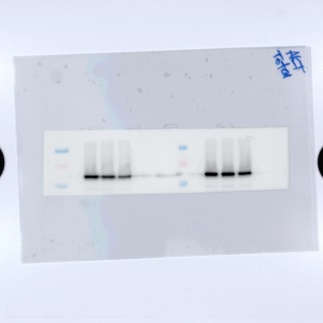


Title of data: Fig. 6A t-Akt-1

Description of data: t-Akt of siRNA treated T24 in Fig. 6A.


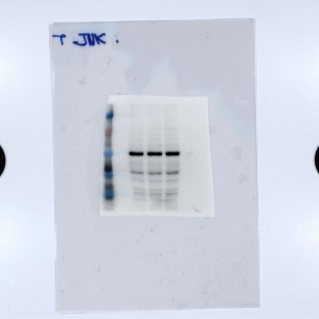


Title of data: Fig. 6A t-JNK-1

Description of data: t-JNK of siRNA treated T24 in Fig. 6A.


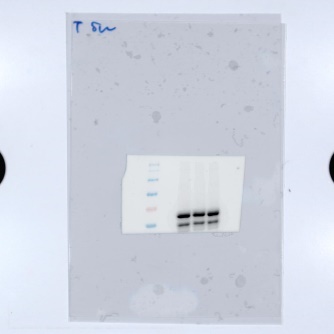


Title of data: Fig. 6A t-Src-1

Description of data: t-Src of siRNA treated T24 in Fig. 6A.


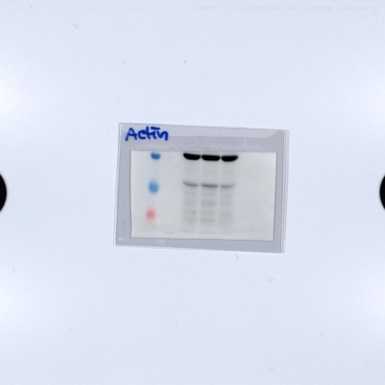


Title of data: Fig. 6A beta-actin-2

Description of data: Beta-actin of siRNA treated UMUC3 in Fig. 6A.


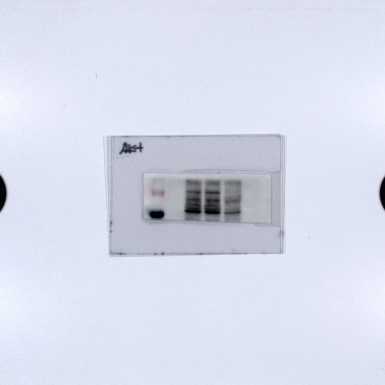


Title of data: Fig. 6A p-Akt-2

Description of data: p-Akt of siRNA treated UMUC3 in Fig. 6A.


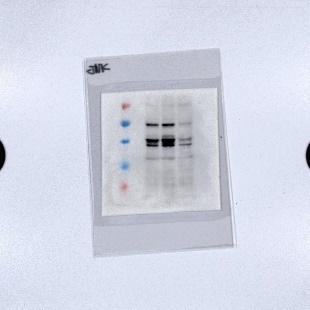


Title of data: Fig. 6A p-JNK-2

Description of data: p-JNK of siRNA treated UMUC3 in Fig. 6A.


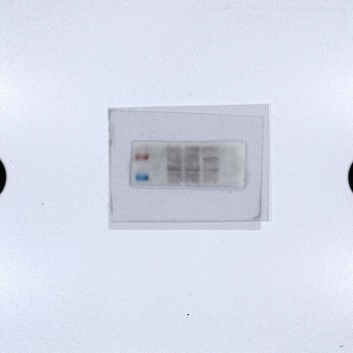


Title of data: Fig. 6A p-Src

Description of data: p-Src of siRNA treated UMUC3 in Fig. 6A.


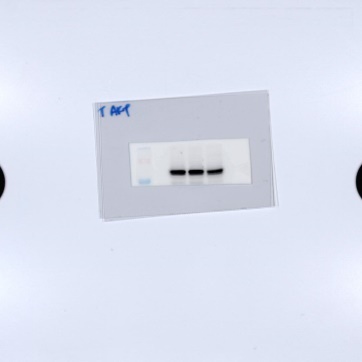


Title of data: Fig. 6A t-Akt-2

Description of data: t-Akt of siRNA treated UMUC3 in Fig. 6A.


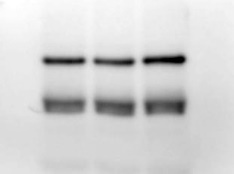


Title of data: Fig. 6A t-JNK-2

Description of data: t-JNK of siRNA treated UMUC3 in Fig. 6A.


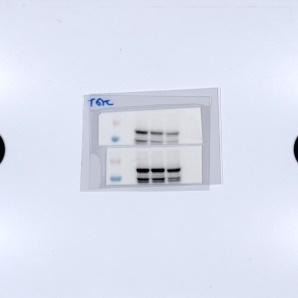


Title of data: Fig. 6A t-Src

Description of data: t-Src of siRNA treated UMUC3 in Fig. 6A.
